# EFFECTS OF A MULTICOMPONENT PHYSICAL ACTIVITY PROGRAM ON STRENGTH AND MOBILITY IN OLDER ADULTS IN CARE HOMES

**DOI:** 10.1093/geroni/igae098.1896

**Published:** 2024-12-31

**Authors:** Yijian Yang, Ziwei Zeng, Jiahao Shen, Cheuk Yin Ho, Ka Po Chan, Francis Kin Tung Chan, Chi Tat Fung

**Affiliations:** Chinese University of Hong Kong, Hong Kong, China (People’s Republic); Chinese University of Hong Kong, Hong Kong, China (People’s Republic); Chinese University of Hong Kong, Hong Kong, China (People’s Republic); Chinese University of Hong Kong, Hong Kong, China (People’s Republic); Chinese University of Hong Kong, Hong Kong, China (People’s Republic); Hong Kong Sheng Kung Hui Welfare Council Limited, Hong Kong, Hong Kong; Hong Kong Sheng Kung Hui Welfare Council Limited, Hong Kong, Hong Kong

## Abstract

Physical activity (PA) is important for maintaining functional mobility in frail older adults. However, existing PA programs for older adults mainly focus on lower limb function but overlook upper limb and core strength, reaction time, and coordination, which are crucial for safe transferring and fall prevention. In this study, we evaluated a multicomponent PA program, Mobility-Fit that we previously developed for frail older adults, through a cluster randomized controlled trial. 164 older adults from 20 care homes in Hong Kong were recruited and randomly assigned into the intervention group (practicing Mobility-Fit 3 times/week, 45 minutes/session for 12 weeks) or a control group that maintained usual care. All participants underwent assessments of strength, mobility, cognition, PA level (measured by accelerometers), and falls efficacy at baseline and completion. Repeated-Measures Linear Models were used to examine changes within and between groups. The mean age of participants was 84.4±8.0 years. 58% were females. Among the intervention group, handgrip strength increased by 15% (p=0.036), knee extension strength increased by 16% (p=0.012), reaction time decreased by 7% (p=0.049), and five-time sit-to-stand duration decreased by 12% (p=0.035). There were significant interactions between time and group in these variables (p < 0.05). Conversely, participants in control group had a 6% decrease in handgrip strength (p=0.043) and higher fall efficacy score (p=0.048). No difference was found in cognitive performance. Our results suggest that multicomponent physical activities are effective in enhancing strength and mobility among frail older adults. The Mobility-Fit program may suit older adults with different functional capacities in care homes.

